# Integrating complementary and alternative medicine into academic medical centers: Experience and perceptions of nine leading centers in North America

**DOI:** 10.1186/1472-6963-5-78

**Published:** 2005-12-20

**Authors:** Sunita Vohra, Kymm Feldman, Brad Johnston, Kellie Waters, Heather Boon

**Affiliations:** 1 CARE Program, Department of Pediatrics, University of Alberta, Edmonton, Alberta, Canada; 2 Department of Family and Community Medicine, University of Toronto, Toronto, Ontario, Canada; 3 Leslie Dan Faculty of Pharmacy, University of Toronto, Toronto, Ontario, Canada

## Abstract

**Background:**

Patients across North America are using complementary and alternative medicine (CAM) with increasing frequency as part of their management of many different health conditions. The objective of this study was to develop a guide for academic health sciences centers that may wish to consider starting an integrative medicine program.

**Methods:**

We queried North American leaders in the field of integrative medicine to identify initial sites. Key stakeholders at each of the initial sites visited were then asked to identify additional potential study sites (snowball sampling), until no new sites were identified. We conducted structured interviews to identify critical factors associated with success and failure in each of four domains: research, education, clinical care, and administration. During the interviews, field notes were recorded independently by at least two investigators. Team meetings were held after each visit to reach consensus on the information recorded and to ensure that it was as complete as possible. Content analysis techniques were used to identify key themes that emerged from the field notes.

**Results:**

We identified ten leading North American integrative medical centers, and visited nine during 2002–2003. The centers visited suggested that the initiation of an integrative medicine program requires a significant initial outlay of funding and a motivated "champion". The centers had important information to share regarding credentialing, medico-legal issues and billing for clinical programs; identifying researchers and research projects for a successful research program; and strategies for implementing flexible educational initiatives and establishing a functional administrative structure.

**Conclusion:**

Important lessons can be learned from academic integrative programs already in existence. Such initiatives are timely and feasible in a variety of different ways and in a variety of settings.

## Background

Complementary and alternative medicine (CAM) has enjoyed substantial growth in recent years [1,2]. A commonly accepted definition of CAM is a "group of diverse medical and health care systems, practices, and products that are not presently considered to be part of conventional medicine [3]."

Several studies have documented the widespread use of CAM in the United States, Canada, Europe, and beyond [4-7]. Until recently, the "typical" user was described as an educated female of upper socioeconomic status. More recent studies suggest CAM use is now common among the majority of health care users, and appears to be greater among those with serious, chronic, or recurrent illness [5,8]. The World Health Organization estimates that CAM is used as first-line therapy by a majority of the world's population [9]. Given the multicultural society and large number of first generation immigrants that many academic medical centers serve, it is likely that many of these patients have explored CAM. At the present time, there is a lack of information regarding how CAM is being integrated into academic medical institutions, if it is being integrated at all [10]. Although many multidisciplinary medical centers aim to treat the "whole person", with consideration given to emotional, social, and environmental factors that may play a part in illness, most programs do not yet integrate CAM therapies even if they have demonstrated efficacy and cost-effectiveness [11,12]. While there are many definitions of "integration", for our purposes it is defined as a collaborative team approach that includes both conventional "Western" and CAM health care providers.

Our intent was to identify successful academic integrative medicine programs and elicit their experiences and perceptions of their own strengths and weaknesses, with a goal of advising the future development of an academic integrative medicine program. The authors of this study sought to answer the questions: how would one go about setting up a new integrative medicine program at an academic health sciences centre? What would be the important tools one would need? Who should be the key individuals involved? To answer these questions, we made a series of site visits to leading integrative medicine programs operating at academic health science centers across North America.

## Methods

Sites were chosen on the basis of their reputation for excellence in a minimum of two of three areas of interest: research, clinical care, and education. Using snowball sampling, we queried North American leaders in the field of integrative medicine, and the key stakeholders at each of the initial sites visited, to identify potential study sites, until no new sites were identified [13]. Interviewees were chosen on the basis of their role at the participating center, and represented leadership from all facets of the program (research, clinical care, education, and administration). A minimum of two investigators attended each site visit (and in three instances, three investigators attended). One investigator attended all site visits (SV). At each centre, questions were asked about the clinical, research, educational and administrative aspects of the integrative medicine program. As well, the participants were interviewed with regards to critical factors for success regarding the team, its resources, structure and process. A summary of the site visit interview guide is provided in Table 1 and a complete version can be obtained by contacting one of the authors (SV).

**Table 1:** Summary of site visit interview guide

***Previsit:***
Request intake forms
List of personnel – name and role
List of resources they utilize/recommend
***Visit:***
1) Background
History/evolution of clinic
Who are components of team
2) Clinical
What range of conditions do you treat?
Is it a centralized service (or local, housed in various divisions)?
Is your clientele of a predominant ethnicity?
How do patients access the clinic?
What intake forms do you use?
Do you provide a consult service only, or continuing care?
Do you recommend specific treatments? On what level of evidence? How is this decided? Do you sell anything (e.g., supplements) on site? Why or why not?
How do you come to decide which products/practices/practitioners to recommend?
Do all team members see all patients or is there a "team leader" who makes those decisions?
How long are initial appointments, follow-ups, and appointments with each team member?
How do team meetings/Rounds work?
3) Research
What research is underway at present? What projects are completed? What did you start with?
4) Education
Are all team members involved in the provision i.e., rounds, clinical activities?
Who comes to your center as a trainee?
Do you provide community education i.e. to the rest of hospital/health center, consumers, etc.?
5) Operations
How much space do you have?
How are you funded?
Is it fee-for-service or salaries for your clinical staff?
Is it covered by insurance, or pay-out-of-pocket?
Has it been a hindrance to have patients pay?
How do you handle legal issues?
6) Advice
What were your critical factors for success/failure?

During the interviews, field notes were recorded independently by the investigators to ensure reliability. Field notes included both the content of answers to the questions in Table 1, as well as details about the environment and interaction between practitioners observed during the visit. Team meetings were held after each visit to reach consensus on the information recorded and to ensure that it was as complete as possible. Interviews were not audio-taped because many took place as part of a "tour" of the site and background noise made recording difficult. Content analysis techniques were used to identify key issues that emerged from the field notes [14,15]. These were discussed at team meetings until consensus was reached. We received permission from each site to make the content of this manuscript public.

## Results

We identified ten leading North American integrative medical centers. Visits took place during 2002–2003 and involved a total of nine sites. We could not visit the tenth site identified due to time constraints. Site visits provided a snapshot of the top integrative academic health science centers in North America. The authors learned how each started their program, how it evolved, what worked, and what did not. The earliest integrative medicine program visited opened in 1991, while the rest launched in the intervening decade. A common reason for the initiation of the CAM programs visited was an underlying interest in the center, catalyzed by a directed donation or endowment fund (usually $1–10 M USD). Each program stressed the importance of having a well-respected person (usually an MD) who could "champion" the initiative. The findings can generally be divided into four domains: 1) clinical programs; 2) research programs; 3) educational programs; and 4) administrative structure.

### Clinical programs

The programs visited varied from the largest provider of integrative care in the US to programs with accessory virtual clinics or programs with clinics that were planned, but did not yet exist. On-site CAM services included Traditional Chinese Medicine (TCM) as a whole system, acupuncture, TCM supplements, chiropractic, massage, aromatherapy, homeopathy, herbal medicine, mind-body and biofeedback. Conventional Western medical care was always offered on-site at the same facility, with services such as family medicine, internal medicine, psychiatry, physiotherapy, nutrition, etc., as well as conventional medical trainees in most sites.

Patients attending academic integrative medicine programs presented with a broad range of complaints including: menopausal symptom management, chronic fatigue, fibromyalgia, depression, irritable bowel syndrome, chronic pain, emotional/mental health, infertility, asthma, and symptoms related to cancer/cancer therapy. Providers at the sites agreed that patients came to the clinic because the program assessed the "whole person", rather than for a second opinion on their diagnosis. While all programs insisted patients see a team physician as part of their assessment (if they were not already under the care of a family physician), the physician was not necessarily the gatekeeper (i.e., did not have to see the patient before the other providers, nor was physician approval to see a particular CAM provider necessary in most centers). Experience has taught the practitioners that "less is more", e.g., having more CAM providers involved in each patients' care was expensive and could leave a patient feeling confused. Instead, the goal was to have a clear and simple care plan, and an opportunity to develop a meaningful relationship with one care provider. Outcomes were reviewed approximately every three months and if the patient was not making progress or achieving their goals, the treatment plan was reviewed.

The different programs visited appeared to interpret the concept of practicing "evidence-based medicine" differently. Given the frequent gaps in the evidence with respect to CAM, a common approach was that if there is evidence that a product or therapy does not work or is harmful, then it was not be used. However, if there was simply inadequate evidence to prove efficacy, many programs were willing to offer it, if only so that it could be studied. One person described their approach as asking: "Does it make sense? Is it reasonably priced?" Decisions were made based on the answers to these questions in the absence of information about efficacy.

Program choices regarding which CAM services to provide at each center were in part based on regulatory status, partly on patient demand, and also on the ability to find the right individuals to become part of the team. The goal was not to train the MD in all modalities of CAM, but to work with CAM experts so that all team members could learn when and how to refer appropriately. When choosing CAM practitioners for the clinic, the sites emphasized the importance of carefully assessing the individuals applying. Several cites voiced it was critical to involve CAM providers who were certified in their own field and who stayed within their scopes of practice in order to minimize liability concerns. In most clinics, all CAM providers carried liability insurance that they paid for themselves (up to $3 M USD). Program directors emphasized that CAM providers who had "cross-training" (i.e., with conventional medical professions) were preferred. The CAM practitioner was advised that for the clinic's purpose, scope of practice may be limited compared to what they could do outside of the program.

As CAM was not covered by many private or public insurers, the programs had to create strategies to overcome potential inequities based on a fee-for-service system. Some programs billed every patient, irrespective of insurance status, leaving it up to the individual to see if their costs will be reimbursed. Others had subsidy programs or reduced fees for patients who could not afford care. Very few programs were profitable, yet they agreed "no margin, no mission," suggesting that above all, it was necessary to stay in business if one wished to help patients.

In almost all circumstances the clinics held a multidisciplinary case conference about patients, the content of which has evolved over time. The intent of this conference was usually to broaden awareness of each team member's knowledge and scope of practice, and to coordinate patient care plans. In most instances, more established programs spent less time on this activity, and in some it was eliminated because it was too expensive or no longer needed (i.e., collaborative relationship and trust had been established and providers were comfortable with referral).

### Research programs

Programs have generally discovered that research was a critical element to success. With evidence, they were better able to convince skeptical colleagues about the value of their approach. Some sites grew around a foundation of research; others were adding it as time passed. One program advised choosing research areas based on where team members have passion, interest and expertise. Another program did a survey of University faculty members and found 400 members who were interested in CAM research. From this, their strategy was to build the center with people from within, thus building confidence and acceptance by engaging local senior researchers. The key was to find internal, open-minded, rigorous clinical and basic science researchers. Another team acknowledged that most of their research actually happened off-site, e.g., with collaborators in nursing, pharmacy, internal medicine and psychiatry.

Different programs had different research foci. One was looking at phase I-III trials in herbal medicine, including basic sciences questions such as mechanism of action, or examination of the immunological effects of whole formula vs. single "active" ingredient *in vitro*. In another center, every client was asked to participate in outcomes research, with measures taken at baseline, six months, and 12 months. The measures were used to try to capture self-reported health concerns, client satisfaction, social support, and pain. The research nurse would meet with all patients when they entered the program, and discuss their goals and motivation for coming to the program. She would contact them throughout the care process, and assessed the six month measures (How are you feeling now? What do you attribute that to?).

### Educational programs

The scope of the educational programs varies from site to site. Most established centers had an education director, and had set up a variety of educational opportunities. These included lectures to and electives for medical students, rotations for internal medicine residents, and a survey course where medical students learn about and experience CAM therapies. The students were required to do research during rotations, usually a literature search and presentation at rounds. At one site, 25 community-based CAM providers donated their time to run workshops. Another center started a fellowship program in 1999, and at the time of our visit, had an NIH training grant for six fellows. They also held an annual continuing medical education (CME) event and prepared online cases for CME credit. A different center had an exchange elective whereby medical students met with students from the American College of Traditional Chinese Medicine to teach each other about physical examination and diagnosis, and "share the process of becoming a healer" in both systems (students teaching students).

### Administrative structure

We queried participating sites on the operations of their integrative program as well as the critical factors to the success/failure regarding the team, its resources, structure and process (see Table 2).

**Table 2:** Key themes for making an integrative medical program work

1) Start small, stay flexible, make as few financial commitments as possible
2) Involve the best (clinical) team members possible (don't compromise, listen to instinct)
3) Keep research focused, but clinic broad
4) Recruit from within where possible (it is expensive to bring people in)
5) Develop benchmarks to evaluate progress
6) Track utilization (who calls, who comes) – useful for reports to funding partners and for grant proposals
7) Hire the team before opening clinic doors
8) Streamline administration
9) Electronic medical records – can save money in long run
10) Recommend good technology/infrastructure that is scalable, with ideally the same firewall as the university or hospital (same IS support, maintenance, etc.)
11) Maximize revenue generating space

The administrative structure varied between programs. Often team members reported to research/education/clinical directors, who reported to a program director, who, in turn, reported to the program advisory board and, in centers affiliated with medical schools, the Dean of Medicine. Advisory board membership was usually chosen to represent a diversity of perspectives as well as opinion leaders across different fields to maximize "buy-in". Typically the advisory board met once a year, while the executive committee (or the directors within the program) met every two weeks to every two months depending on the number of issues to discuss. All the staff of the center might meet quarterly for one hour (often at lunchtime) for strategic updates from the executive committee.

Duties of the clinic director were to oversee such things as credentialing, malpractice issues, pharmacy and therapeutics committee, and clinical care delivery. The research director oversaw all research associated with the program and was often responsible for obtaining research funding. The education director would typically oversee trainee rotations, fellowship programs, CME events, and be a liaison to the medical school. Most program directors were responsible for overseeing clinic administration, such as finances, human resources, payroll, contracts, and the day-to-day operations of the clinic etc. Some clinics also had a fundraising committee. The key themes for the administration of a successful integration program are listed in Table 2.

## Discussion

Integration of CAM research, education, and clinical care delivery in academic health science centers is occurring in many US institutions. Canadian initiatives are few in number, and limited in scope. Given the increasing demand for CAM services, this is an important area for future growth in all North American medical centers. The existing programs have important information to share regarding credentialing, medico-legal issues and billing for clinical programs; identifying researchers and research projects for a successful research program; and strategies for implementing educational initiatives and establishing a functional administrative structure. It is important to note that most of the centers visited do not yet have all three "pillars" of clinical care, research and education, with various reasons for this. Some started with clinical care, others will add that last. Most have learned that research and education are important components and are working to expand these areas within their programs.

We demonstrate that CAM has been successfully integrated in nine North American academic medical centers. Centers displayed diverse implementation strategies and had made different arms (research, clinical and education) operational at different times. Some had a separate integrative hospital-based clinic consisting of CAM and conventional providers for broad patient care needs; others had a narrow, highly specialized patient care approach, such as for acute and chronic pain (e.g., dental pain, osteomyelitis, rheumatoid/osteoarthritis, etc.). Each site varied in available funding and the strength of its affiliation to a University.

An integrative medicine program fits with the core values and beliefs of many academic medical centers. For example, 29 renowned academic medical institutions in North America have recently joined to form The Consortium of Academic Health Centers for Integrative Medicine [16]. Their mission is to help advance medicine and healthcare through rigorous scientific studies, new models of clinical care, and innovative educational programs that integrate biomedicine, the complexity of human beings, the intrinsic nature of healing and the rich diversity of therapeutic systems.

While advocates of integrative medicine may speak of "evidence-based" CAM [17]; integration within academic medical centers may foster double standards for the inclusion of CAM therapies [18]. For instance, "evidence-based medicine" for some narrowly refers to the practice of taking randomized controlled trials as the strongly preferred form of evidence for medical practice; however, we observed that each of the integrative clinical programs have, like most of conventional medicine, taken care to allow a role for other factors (i.e., clinical experience, clinical state and circumstances of patient, patient preference) beyond evidence from randomized controlled trials [19,20]. Indeed, "as a distinctive approach to patient care, EBM involves two fundamental principles. First, evidence alone is never sufficient to make a clinical decision. Decision makers must always trade the benefits and the risks, inconvenience, and costs associated with alternative management strategies, and in doing so consider the patient's values. Second, EBM posits a hierarchy of evidence to guide clinical decision making" [21]. The fundamentals of "integration" will sometimes require clinicians to ignore unbridgeable epistemological practices and beliefs between conventional and CAM [18].

Our study has three major limitations. First, while every attempt was made to engage the centers to allow us to sit in on a case conference, unfortunately this was not possible. The centers that had been in existence for the longest period of time no longer held them, although they agreed they were an important component for sites new at providing integrative care. The centers that were more recently formed still held these team meetings, but did not give permission for outside observers to attend. Therefore, we were not able to directly observe the functioning of the multidisciplinary teams. However, one of us (KF) previously trained for a short period of time at a site which held case conferences and was therefore able to contribute that experience to our larger understanding. Second, one of ten leading integrative programs in North America was not assessed due to time constraints. This may have led to the omission of further potentially useful information, although we had already reached saturation from the nine sites visited. Of note, our intent was not to identify specific subspecialty programs (e.g. integrative oncology centers); thus our findings may not be generalizable to such centers. Third, one of the investigators (SV) intended to, and now has, set up an integrative medical center at an academic institution which may have biased the collection and interpretation of interview data. Having two investigators independently record and interpret field notes at each site helped to both ensure reliability and overcome this limitation.

Our study also had several strengths. As far as we are aware, this is the first qualitative analysis of the experiences and perceptions of site leaders (e.g. clinical directors, research directors) at multiple integrative academic medical centers. The key themes we present should be used to help guide academic centers wishing to integrate CAM. Our team was multidisciplinary in its research and clinical expertise and reflected a variety of points of view (e.g. health services research, qualitative research, family medicine, pediatrics, pharmacy as well as naturopathy). Our recommendations are based on the experiences of nine of ten highly recommended integrative medical centers in North America, and have been applied in the development of Canada's first academic pediatric integrative medicine program (CARE Program, Stollery Children's Hospital, Edmonton, Canada).

CAM is a widespread patient driven initiative. Academic institutions have an opportunity to develop new initiatives in integrative medicine that could help healthcare providers and patients meet their information needs, enable more evidence-based decision-making, facilitate the development of new knowledge, and enhance health outcomes. For academic centers wishing to build such initiatives, our findings illustrate integration is timely and feasible in a variety of different ways and in a variety of settings.

## Competing interests

The authors declare that they have no competing financial interests. SV leads an academic integrative medical program in Edmonton, Canada.

## Authors' contributions

SV conceived the study and developed the site visit questionnaire in collaboration with KF and HB. SV participated in all site visits, took field notes, wrote the first draft of the original report, and guided manuscript revisions. KF took field notes at eight sites, participated in the first draft of the original report and participated in revisions of manuscript. BJ and KW drafted the initial manuscript for journal submission and participated in revisions. HB provided qualitative methods expertise, coordinated the design, and analysis of the study, took field notes at six sites and assisted in revisions of the manuscript. All authors read and approved the final manuscript.

## Pre-publication history

The pre-publication history for this paper can be accessed here:


